# Effects of Oral Lycopene Supplementation on Vascular Function in Patients with Cardiovascular Disease and Healthy Volunteers: A Randomised Controlled Trial

**DOI:** 10.1371/journal.pone.0099070

**Published:** 2014-06-09

**Authors:** Parag R. Gajendragadkar, Annette Hubsch, Kaisa M. Mäki-Petäjä, Martin Serg, Ian B. Wilkinson, Joseph Cheriyan

**Affiliations:** 1 Clinical Pharmacology Unit, University of Cambridge, Cambridge, United Kingdom; 2 Department of Cardiology, University of Tartu, Tartu, Estonia; 3 Cambridge Clinical Trials Unit, Cambridge University Hospitals National Health Service Foundation Trust, Cambridge, United Kingdom; 4 Cambridge University Hospitals National Health Service Foundation Trust, Cambridge, United Kingdom; Indiana University Richard M. Fairbanks School of Public Health, United States of America

## Abstract

**Aims:**

The mechanisms by which a ‘Mediterranean diet’ reduces cardiovascular disease (CVD) burden remain poorly understood. Lycopene is a potent antioxidant found in such diets with evidence suggesting beneficial effects. We wished to investigate the effects of lycopene on the vasculature in CVD patients and separately, in healthy volunteers (HV).

**Methods and Results:**

We randomised 36 statin treated CVD patients and 36 healthy volunteers in a 2∶1 treatment allocation ratio to either 7 mg lycopene or placebo daily for 2 months in a double-blind trial. Forearm responses to intra-arterial infusions of acetylcholine (endothelium-dependent vasodilatation; EDV), sodium nitroprusside (endothelium-independent vasodilatation; EIDV), and NG-monomethyl-L-arginine (basal nitric oxide (NO) synthase activity) were measured using venous plethysmography. A range of vascular and biochemical secondary endpoints were also explored. EDV in CVD patients post-lycopene improved by 53% (95% CI: +9% to +93%, *P* = 0.03 vs. placebo) without changes to EIDV, or basal NO responses. HVs did not show changes in EDV after lycopene treatment. Blood pressure, arterial stiffness, lipids and hsCRP levels were unchanged for lycopene vs. placebo treatment groups in the CVD arm as well as the HV arm. At baseline, CVD patients had impaired EDV compared with HV (30% lower; 95% CI: −45% to −10%, *P* = 0.008), despite lower LDL cholesterol (1.2 mmol/L lower, 95% CI: −1.6 to −0.9 mmol/L, *P*<0.001). Post-therapy EDV responses for lycopene-treated CVD patients were similar to HVs at baseline (2% lower, 95% CI: −30% to +30%, *P* = 0.85), also suggesting lycopene improved endothelial function.

**Conclusions:**

Lycopene supplementation improves endothelial function in CVD patients on optimal secondary prevention, but not in HVs.

**Trial Registration:**

ClinicalTrials.gov NCT01100385

## Introduction

The incidence of cardiovascular disease (CVD) varies worldwide but is notably reduced in southern Europe where a ‘Mediterranean diet’ predominates; consisting mainly of a larger consumption of fruit, vegetables and olive oil [1], [2]. Recent primary prevention interventional dietary trials, and a large observational analysis demonstrate that this diet reduces the incidence of CVD events in asymptomatic patients at high cardiovascular risk, and also, in conjunction with effective secondary prevention medication, is associated with a lower incidence of recurrent CVD events [3]–[5]. The mechanisms underlying this effect are unclear but may be partly due to a high antioxidant component of the diet.

Lycopene is a lipophilic, active carotenoid component of tomatoes giving them their distinctive red colour. It is a potent antioxidant with a singlet-oxygen quenching ability twice that of β-carotene and ten times that of Vitamin E due to its structure (**Figure S1**) [6]. There is an inverse association between lycopene levels and surrogate endpoints of cardiovascular disease in observational studies, and *in-vitro* studies have suggested anti-atherogenic mechanisms of action [7]–[12]. Interventional studies investigating *in-vivo* effects on vascular function have been limited by utilisation of unstandardized food based interventions involving consumption of large volumes of tomato products (e.g. juice or paste) or by the use of heterogeneous tomato extracts containing mixtures of carotenoids [13]–[19].

The ‘residual risk’ of developing further events seen in some CVD patients, despite aggressive lipid lowering with statins and other drugs, may be partly explained by persistent underlying impaired endothelial function [20]–[22]. Nitric oxide (NO) acts as a central signal transduction pathway in the endothelium, regulating haemostasis and platelet aggregation as well as vascular tone [20], [23], [24]. Diminished NO levels are seen in early in atherosclerosis [20]. NO bioavailability can be assessed *in-vivo* by venous occlusion plethysmography, using intra-arterial infusion of acetylcholine, which prospectively predicts risks of developing CVD related events and improves risk classification beyond the Framingham scores [25], [26].

We hypothesised that lycopene would improve endothelial function in patients with pre-existing CVD, and separately, in healthy volunteers (HV) also. In order to determine lycopene’s mechanistic effects on vascular physiology, we used a commercially available oral lycopene preparation with high bioavailability (Ateronon, Cambridge Theranostics, UK). Endothelial function was determined using forearm vascular responses to acetylcholine (ACh; stimulating NO production). Secondary outcomes included forearm responses to sodium nitroprusside (SNP; measuring vascular smooth muscle sensitivity) and N^G^-monomethyl-L-arginine (L-NMMA; measuring basal NO synthase activity) infusion, arterial stiffness, blood pressure, serum lycopene concentrations, and safety and tolerability parameters. A number of exploratory end-points including oxidised low-density lipoprotein (ox-LDL), high sensitivity C-reactive protein (hsCRP), cytokine profile, urinary isoprostanes and plasma nitrotyrosine levels (markers of oxidative stress) were measured to explore postulated mechanisms.

## Methods

### Study Design and Ethics Statement

**Figure 1:**
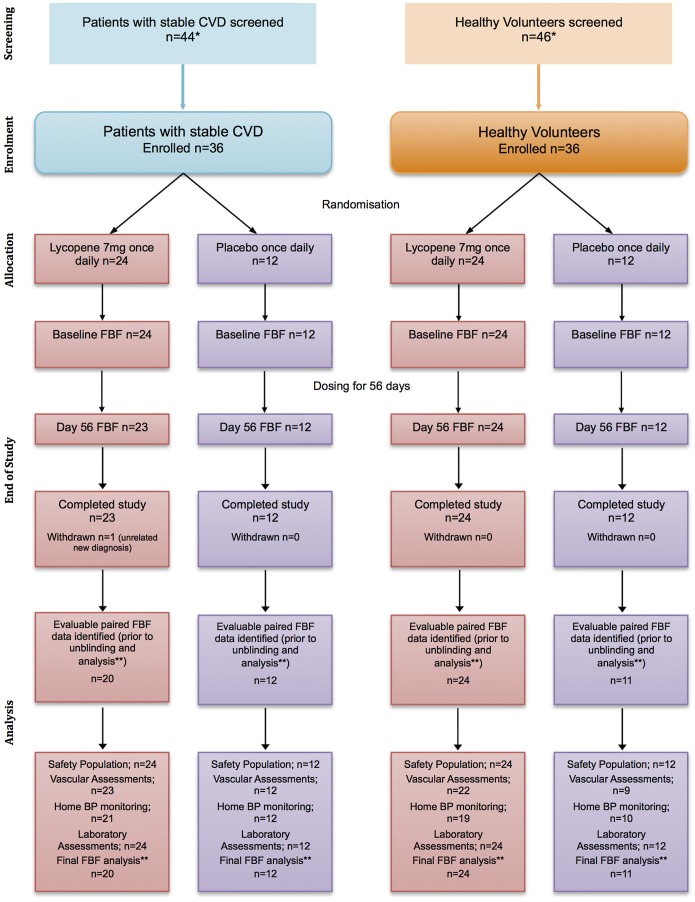
Flow diagram of subjects through the study. The safety population consisted of anyone who received at least 1*Reasons for failure to enrol included not meeting inclusion criteria, an inability to attend laboratory for assessments within the appropriate timeframe, patient withdrawal, inability to lie flat for a period of time for the studies, or an inability to cannulate the brachial artery. **Quality control evaluation done by two independent parties. Reasons for non-evaluable data and consequent exclusion from final forearm blood flow (FBF) analysis (before unblinding and statistical analysis) included incomplete data sets, non-evaluable sets, and FBF procedure variation.

This was a prospective, randomised, double-blind, placebo-controlled, parallel group study comparing lycopene 7 mg with placebo (Ateronon and matching placebo, Cambridge Theranostics, Cambridge, UK) in two separate arms, namely CVD patients and HVs (**Figure 1**). The study was conducted in the Clinical Pharmacology Unit, University of Cambridge, United Kingdom. The protocol received a favourable opinion from the Hertfordshire Research Ethics Committee, and was deemed not to be a clinical trial of an investigational medical product by the Medicines and Healthcare products Regulatory Agency (MHRA). As the trial was a physiological study, there was no requirement for it to be registered on a clinical trials database based on UK regulations in 2009. Nevertheless, the trial was registered at clinicaltrials.gov (NCT01100385) approximately two weeks after commencement, and complied with the Declaration of Helsinki with full written informed consent from all subjects.

The protocol for this trial and supporting CONSORT checklist are available as supporting information; see **Protocol S1** and **Checklist S1**.

### Study Populations

There were two separate, yet parallel arms to our study – patients with CVD and separately, HVs.

In our CVD arm, patients aged 40–80 with stable cardiovascular disease (defined as any one or more of previous myocardial infarction, coronary stent, angina diagnosed on angiography/other imaging modality or exercise/stress testing, transient ischaemic attack or stroke disease, or peripheral vascular disease) were recruited if they were on stable statin therapy for at least two months. Exclusion criteria were uncontrolled hypertension of >180/110 mmHg, a Body Mass Index (BMI) >35 kg/m^2^, pregnancy, or active malignancy. CVD patients were asked to continue on their regular medications, without changes, throughout the trial period.

Healthy volunteers aged 30–80 with no smoking history were eligible for a separate, parallel arm of the study. Subjects in this arm were also required to be normotensive, non-diabetic, not pregnant, and on no regular medication including the oral contraceptive pill, nor statins or other vasoactive drugs including Non-Steroidal Anti-Inflammatories (NSAIDs). Volunteers with a BMI >32 or <18 were excluded as were those with active renal, respiratory, neurological, or oncological disease.

We did not attempt to prospectively stratify each arm to the other since these were parallel studies.

### Interventions

Lycopene 7 mg and placebo (Ateronon and matching placebo in shape, size and colour) were donated by Cambridge Theranostics (Cambridge, United Kingdom). Drug and placebo were randomised unequally (2∶1 ratio) by the independent manufacturer of Ateronon (Indena, Milan, Italy). Subjects in each study arm (CVD or HV) were sequentially allocated from a computer-generated randomisation to receive either lycopene 7 mg or placebo once daily for two months. Study personnel and subjects were blinded to treatment assignment for the duration of the study and final analysis of data. Subjects were advised to maintain their regular diet without restrictions or significant changes to replicate ‘real world’ conditions, more so since we were measuring lycopene levels at study start and end in all subjects.

### Forearm Blood Flow

**Figure 2:**
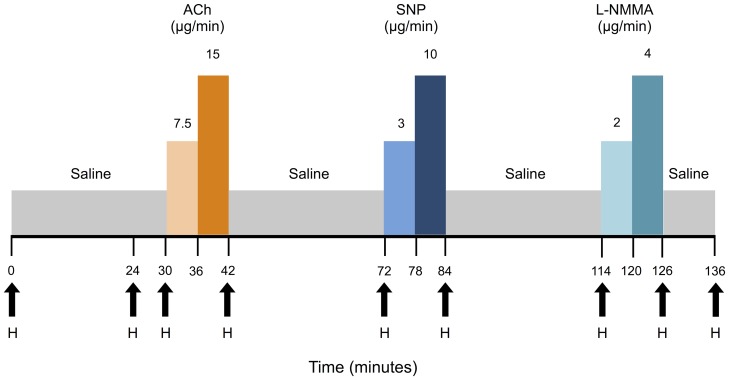
Schematic of forearm blood flow protocol. ACh: Acetylcholine; SNP: Sodium Nitroprusside; L-NMMA: N^G^-monomethyl-L-arginine; H: Haemodynamic measurements.

Forearm blood flow (FBF) was measured by venous occlusion plethysmography (Hokanson Inc, Bellevue, USA) as previously described [25] using the protocol illustrated in **Figure 2**. Wrist circulation was excluded by inflating wrist cuffs above the systolic blood pressure. Upper arm cuffs were intermittently inflated (to 40 mm Hg) and deflated at short intervals over 3 minutes to measure FBF with mercury-in-silastic gauges. The dominant arm was established, where possible, as a control arm without cannulation or test infusions. In contrast, acetylcholine (ACh; Novartis Pharmaceuticals, Basel, Switzerland), sodium nitroprusside (SNP; Nitroprussiat FIDES, Madrid, Spain), and N^G^-monomethyl-L-arginine (L-NMMA; Bachem Distribution Services GmBH, Weil am Rhein, Germany) were infused in a fixed order into the brachial artery of the non-dominant (test) arm via a 27-gauge needle inserted under local anaesthesia. All drugs were prepared aseptically and diluted in sterile saline (0.9% Maco Pharma, London, United Kingdom). All infusions were performed at a rate of 1 mL/min. Saline was infused to establish a baseline before infusion of each challenge agent of acetylcholine (7.5 µg/min; 15 µg/min), SNP (3 µg/min; 10 µg/min), and L-NMMA (2 µg/min; 4 µg/min) (**Figure 2**). Each challenge agent was infused at 2 doses, and each dose was infused for 6 minutes. FBF was recorded in both arms over the last 3 minutes of each infusion.

The primary end-point for each arm of our study was change in endothelium-dependent vasodilatation (EDV; response to 15 µg/min ACh) from baseline, comparing lycopene with placebo treatment allocation groups within each arm. This was chosen based on previous data suggesting that only responses to higher doses of ACh correlated with clinical outcomes [26]. Secondary end-points were endothelium-independent vasodilatation (EIDV; response to 10 µg/min SNP) and vasoconstrictor responses to 4 µmol/min L-NMMA.

Haemodynamics (blood pressure and heart rate) were measured in the brachial artery of the dominant, non-infused arm at baseline and at the end of the infusion period for each challenge agent with a validated oscillometric machine (Omron HEM-705CP, Omron Corp, Kyoto, Japan) [27]. The measurements were taken after 24 minutes and then at the end of each challenge period (**Figure 2**).

Measurements were taken pre-dose on day 1 (baseline), and post-dose on day 56 for all subjects. All measurements were conducted in the morning in a quiet, temperature-controlled (22°C to 24°C) clinical laboratory. Subjects fasted overnight and abstained from alcohol and caffeine-containing drinks for 24 hours before measurement. CVD patients were asked to omit their medications on the morning of the vascular studies. At the end of the whole study, all the FBF data sets underwent quality assessment by two independent blinded parties where any non-evaluable and incomplete data sets were removed from the database before subsequent unblinding and statistical analysis.

### Arterial Stiffness

Measurements of arterial stiffness were conducted as previously described [28]. After 15 minutes of supine rest, peripheral blood pressure was recorded in the brachial artery (OMRON-705CP; Omron Corp, Kyoto, Japan). Aortic (carotid to femoral) pulse wave velocity (PWV) was measured using a high-fidelity micromanometer (SPC-301; Millar Instruments, Houston, USA), and a corresponding central waveform using a validated transfer function (Sphygmocor; AtCor Medical, Sydney, Australia). Augmentation index (AIx) and heart rate were determined with the integrated software. All measurements were made in duplicate and mean values used in the subsequent analyses.

### Blood Pressure

Subjects were asked to record resting, seated home blood pressure (BP) readings both morning and evening using a validated device for any seven days in the 2 weeks prior to vascular assessments. Three readings were made each time, with the average of the final two recorded in a diary and used for analysis. Clinic peripheral BP measurements were taken prior to the arterial stiffness measurements and central BP measurements were estimated non-invasively by the validated Sphygmocor apparatus [29], [30].

### Laboratory Assessments

Blood samples were taken for routine haematology and clinical chemistry tests on day 1 and 56. Samples were also taken for lipid profile, high sensitivity C-reactive protein (hsCRP), oxidised low-density lipoprotein (ox-LDL, Mercodia, Uppsala, Sweden), serum lycopene levels (high-performance liquid chromatography with ultraviolet detection) as well as other exploratory biomarkers. Urine samples were stored immediately at −80°C for urinary isoprostanes (Cell BioLabs Inc., San Diego, USA).

### Safety Assessments

A detailed collection of safety data, including bloods, adverse events and serious adverse events, were monitored throughout the study in accordance with Good Clinical Practice. A complete set of safety observations, including heart rate, blood pressure, and 12-lead ECGs, were recorded at screening, day 1 and 56.

### Statistical Methods

For sample size calculation, the CVD and HV arms were treated as individual studies. Sample size calculation was based on the variability of primary endpoint - change in infused arm forearm responses following 15 µg/min ACh from the preceding saline baseline. Based on a standard deviation of 25% in change from baseline FBF [31], it was estimated that for each arm of our study (i.e. CVD and HV) a sample size of 30 subjects in an unequal 2∶1 randomisation (drug: placebo) would provide 90% power to detect a clinically relevant 20% absolute difference [26] between the groups of change in ACh responses from baseline with a two-tailed alpha level of significance of 5%. The primary endpoint was evaluated separately for CVD and HV arms. An unequal randomisation method was employed for feasibility purposes of conducting such a large study within one academic centre with this robust but minimally invasive technique. We recruited 36 subjects each for the CVD and HV arms (total n = 72 overall) to account for potential dropouts and unevaluable data. Data were analysed on an intention-to-treat basis on the full analysis set of an intention-to-treat protocol as set out by the ICH E9 guidelines on statistical principles for clinical trials [32].

Absolute infused arm FBF values were analysed by infusion agents using a repeat measures analysis of variance model (ANOVA), with a term for drug/placebo, visit day, infusion dose within day, and interaction of treatment and dose within day, in which the preceding saline baseline was treated as infusion dose zero and the higher dose of challenge agent as infusion dose 1. Greenhouse – Geisser corrected probability values were used if Mauchly’s test revealed a violation of sphericity. Blood pressures were averaged from subject diary cards and mean values compared using repeat measures ANOVA with a term for drug/placebo, visit day, and interaction of treatment and day. Home blood pressure variability was calculated by taking the standard deviation of the readings from the subject diary cards prior to each visit and analysing them using repeat measures ANOVA with a term for drug/placebo, visit day, and interaction of treatment and day. Changes in arterial stiffness and concentrations of biomarkers were analysed using analysis of covariance (ANCOVA) with baseline value at day 1 as a covariate and treatment group as fixed factors [33].

Post-hoc comparisons at single time points of baseline physiological and biochemical parameters between healthy volunteers and CVD patients were performed using unpaired, 2-tailed Student *t* tests, or using χ^2^ tests for categorical variables. As a single dose of lycopene was used, variations in serum lycopene levels due to dietary intake in addition to the intervention (lycopene or placebo) were compared to the primary endpoint in a post-hoc analysis to demonstrate dose-response characteristics and to examine any effects of extraneous dietary changes. Correlations between change in lycopene levels and change in FBF responses were investigated by calculating the Pearson product moment correlation coefficient. Adverse event rate was calculated as number of subjects experiencing adverse events in lycopene or placebo groups divided by total numbers of subjects in lycopene or placebo groups, expressed as a percentage, with comparisons made using a χ^2^ test. For all analyses, a probability of <0.05 was considered significant. Statistical analyses were performed using SPSS version 20 (IBM, Somers, New York, USA).

## Results

The study protocol was approved in April 2010 and the final subject completed the study in May 2012 (**Figure 1**).

### Baseline Demographics

The demographics of the lycopene and placebo treatment allocation groups for the separate CVD and HV arms are described in **Table 1**. In general, lycopene and placebo treatment groups were well matched across major variables in both CVD and HV arms. There were slightly more women in the HV arm compared to the CVD arm, reflecting the increased prevalence of cardiovascular disease in men. All CVD patients were on stable doses of statins (mean equivalent simvastatin dose of 40 mg), with a high proportion on other secondary prevention medication such as anti-platelets and anti-hypertensives (**Table 1**).

**Table 1:** Baseline Demographics of CVD Patients and HV arms.

	CVD Patients		HVs	
	Lycopene	Placebo	Lycopene	Placebo
N	24	12	24	12
Age – years [mean (SD)]	67 (6)	68 (5)	61 (13)	68 (5)
Sex - M:F	23∶1	10∶2	15∶9	10∶2
BMI – kg/m^2^ [mean (SD)]	28.6 (3.3)	28.4 (4.0)	25.2 (2.8)	26.7 (3.6)
Current Smoker [n (%)]	2 (8)	0 (0)	0 (0)	0 (0)
Ex-Smoker [n (%)]	18 (75)	6 (50)	9 (38)	7 (58)
EtOH units/week [mean (SD)]	12.9 (11.3)	9.2 (9.1)	11.8 (12.9)	7.6 (7.5)
Statins [n (%)]	24 (100)	12 (100)	0 (0)	0 (0)
ACE-I/ARB [n (%)]	22 (92)	8 (67)	0 (0)	0 (0)
β-blockers [n (%)]	13 (54)	5 (42)	0 (0)	0 (0)
Antiplatelet [n (%)]	24 (100)	12 (100)	1 (4)	0 (0)
Mean duration of dosing – days [mean (SD)]	57 (4)	56 (6)	60 (9)	61 (6)

Data are presented as mean (standard deviation - SD) or numbers (%). ACE-I: Angiotensin Converting Enzyme Inhibitor; ARB: Angiotensin Receptor Blocker; CVD: cardiovascular disease; EtOH: Alcohol; HV: healthy volunteer.

### Forearm Blood Flow

**Figure 3:**
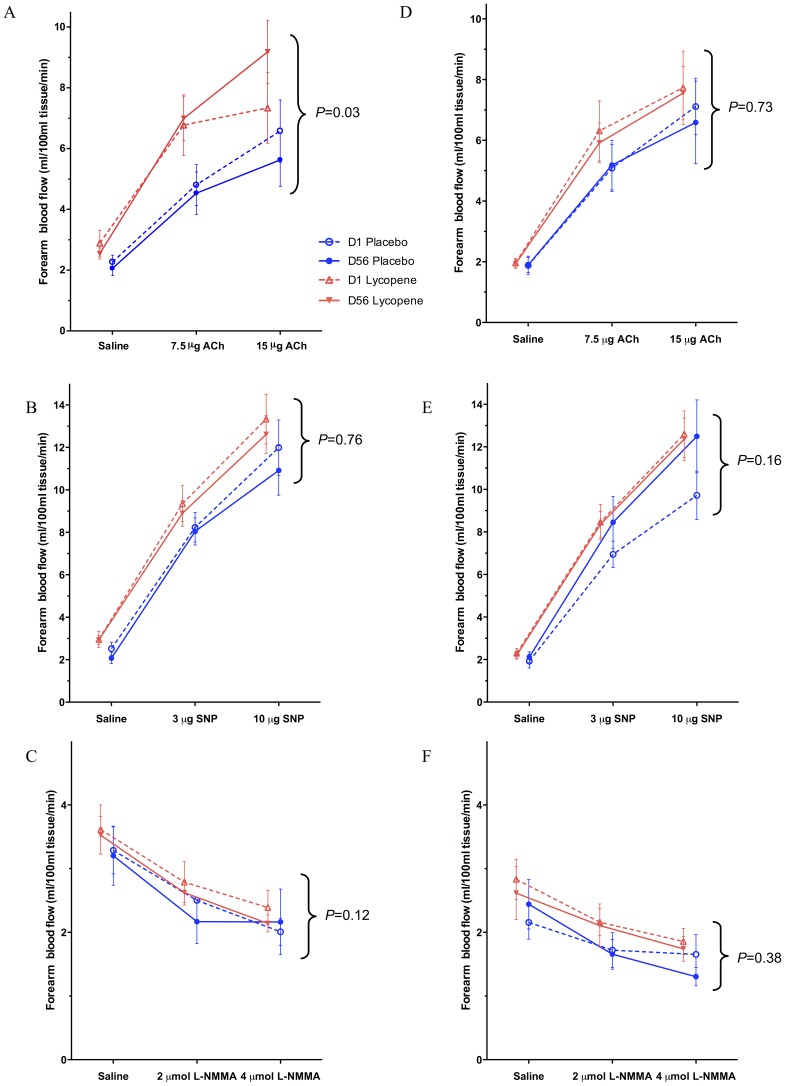
Changes in Forearm Blood Flow. Infused arm forearm blood flow values in cardiovascular disease patients (A–C) and healthy volunteers (D–F) before dose on day 1 (broken lines) and after dose on day 56 (solid lines) for lycopene (red lines) and placebo (blue lines) in response to acetylcholine (ACh; graphs A and C); sodium nitroprusside (SNP; graphs B and D), and N^G^-monomethyl-L-arginine (L-NMMA; graphs C and F) infusions. Values represent mean with standard error (SE) bars. Comparisons were made using a repeat measures ANOVA with terms for drug/placebo, visit day, infusion dose within day, and interaction of treatment and dose within day, in which baseline saline was treated as infusion dose zero. *P*-values presented are for lycopene vs. placebo overall for the higher dose challenge agent.

CVD patients randomised to lycopene treatment demonstrated improved EDV (63% higher, 95% CI: +19% to +108%, *P = *0.008). No changes were noted in EIDV or basal NO synthase activity. No changes were observed over the treatment period in the placebo group (EDV: 7% lower, 95% CI: −41% to +56%, *P* = 0.8); (**Figure 3**; A, B, C). After placebo correction, EDV was improved significantly by 53% (95% CI: +9% to +93%, *P* = 0.03) in the lycopene treated CVD group. No significant differences were seen between lycopene-treated and placebo groups in forearm responses to SNP or L-NMMA (**Table S1**). There were no changes in the control arm FBF values during the challenge agent infusions.

No significant changes were noted in FBF responses to ACh, SNP, or L-NMMA in the HV cohort between lycopene and placebo (**Figure 3**; D, E, F and **Table S2**). There were no changes in the control arm FBF values during the challenge agent infusions.

**Figure 4:**
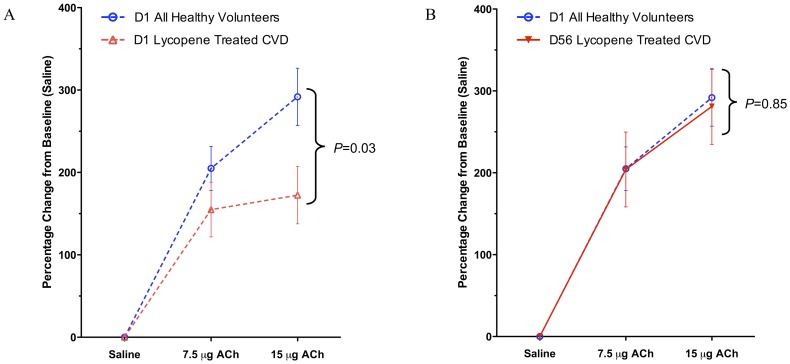
Post-hoc analysis of infused arm FBF values in response to ACh. Forearm blood flow (FBF) values are represented as percentage change from preceding saline baseline with standard error bars. *P*-values were generated from comparisons made using unpaired, 2 tailed Student *t*-tests. (A) At the start of the study, patients with cardiovascular disease (CVD) in the lycopene group (broken red line) had significantly impaired vasodilatory responses to acetylcholine (ACh; 30% lower, 95%CI: −58% to −3%, *P* = 0.03) compared with healthy volunteers (HVs) at baseline (broken blue line). (B) After treatment with lycopene, the same patients (solid red line) show no significant changes in FBF values compared with HVs at baseline (broken blue line) (2% lower, 95% CI: −30% to +30%, *P* = 0.85).

**Figure 5:**
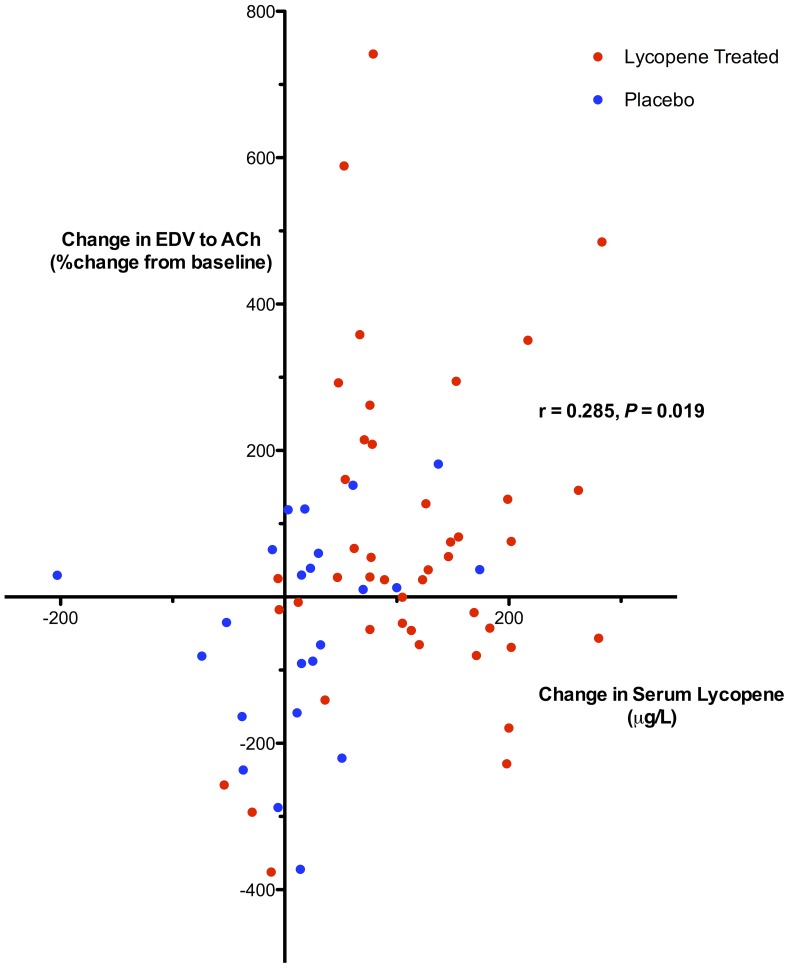
Post-hoc correlation between serum lycopene concentrations and EDV. Relationship between absolute change in serum lycopene concentrations and absolute change in endothelial dependent vasodilatation (EDV; forearm blood flow response to 15 µg/min acetylcholine measured as %change from preceding saline baseline) for all trial subjects. Absolute change in serum lycopene calculated as final visit serum lycopene minus baseline serum lycopene. Absolute change in EDV calculated as final visit EDV minus baseline EDV. r: correlation coefficient calculated using Pearson correlation analysis.

At baseline, CVD patients had significantly impaired EDV compared with HVs (30% lower, 95% confidence interval [CI]: −50% to −8%, *P* = 0.008). No differences were seen in baseline EIDV or vasoconstrictor responses to L-NMMA. In a post-hoc analysis, EDV responses post-lycopene therapy in CVD patients approximated the EDV responses seen in HVs at baseline (2% lower, 95% CI: −30% to +30%, *P* = 0.85), consistent with the relative improvement in endothelial function seen in the lycopene treated CVD arm (**Figure 4** **)**. Post-lycopene EDV responses did not differ between CVD and HVs (16% lower, 95% CI: −28% to +60%, *P = *0.47). In further post-hoc testing to demonstrate dose response characteristics, data from all subjects in both CVD and HV arms of the study were pooled. There was a significant positive correlation between change in lycopene concentration and absolute change in EDV response between visits (r = 0.29, 95% CI: 0.05–0.49, *P* = 0.019, **Figure 5**).

### Arterial Stiffness

There were no changes in arterial stiffness parameters between lycopene treated groups and placebo in CVD patients or HVs (**Tables 2** and **3**). In post-hoc analysis, unsurprisingly at baseline, CVD patients had stiffer arteries compared with HVs as measured by aortic PWV and augmentation index (**Table 4**).

**Table 2:** Vascular and Laboratory Assessments in CVD Patients Arm.

	Placebo		Lycopene		*P*-value*
	Day 1[Mean Value (SE)]	Day 56[Mean Value (SE)]	Day 1[Mean Value (SE)]	Day 56[Mean Value (SE)]	
**Arterial Stiffness**					
Aortic PWV – m/s	8.6 (1.1)	8.1 (1.0)	8.6 (0.3)	8.8 (0.4)	0.2
AIx – %	28.2 (3.1)	26.4 (3.2)	28.7 (1.6)	28.8 (1.5)	0.4
**Blood Pressure**					
Home SBP – mmHg	125 (3)	123 (3)	126 (3)	127 (3)	0.2
Home DBP – mmHg	72 (2)	71 (2)	78 (2)	78 (2)	0.1
Home SBP variability	9.1 (0.7)	9.4 (1)	9.0 (0.7)	8.3 (0.5)	0.2
Clinic SBP – mmHg	138 (3)	137 (4)	137 (3)	133 (3)	0.4
Clinic DBP – mmHg	76 (2)	77 (2)	81 (2)	78 (2)	0.3
Central SBP – mmHg	129 (4)	127 (4)	130 (3)	126 (3)	0.6
Central DBP – mmHg	77 (2)	78 (3)	82 (2)	79 (2)	0.2
**Laboratory Markers**					
Lycopene – µg/L	128 (26)	178 (31)	146 (14)	275 (22)	0.003
LDL – mmol/L	2.41 (0.15)	2.16 (0.14)	2.41 (0.14)	2.41 (0.12)	0.1
HDL – mmol/L	1.48 (0.14)	1.47 (0.16)	1.20 (0.05)	1.17 (0.06)	0.7
hsCRP – mg/L	1.45 (0.59)	1.68 (0.60)	2.13 (0.48)	2.37 (0.58)	0.9
ox-LDL – U/L	31.9 (17.7)	31.8 (15.6)	34.8 (17.2)	36.5 (11.7)	0.3
MMP-9– ng/ml	49.8 (21.3)	44.0 (19.5)	40.3 (17.7)	53.8 (41.1)	0.1
IL-6– pg/ml	1.20 (1.05)	0.92 (0.60)	1.54 (1.31)	1.51 (0.99)	0.3
TNF-α – pg/ml	5.55 (2.95)	5.65 (2.79)	2.13 (0.48)	2.37 (0.58)	0.9
Nitrotyrosine – µM	41.1 (47.4)	112.9 (72.1)	35.0 (20.5)	112.0 (40.9)	0.7

Data are presented as mean values (standard error - SE). *P-value is for overall comparison in delta (day 56 - day 1) values across placebo and lycopene treated groups. AIx – augmentation index; CVD: cardiovascular disease; DBP – diastolic blood pressure; HDL – high-density lipoprotein; hsCRP – high sensitivity C-reactive protein; IL – interleukin; LDL – low-density lipoprotein; MMP-9– matrix metalloproteinase; ox-LDL – oxidised low-density lipoprotein, PWV – pulse wave velocity; SBP – systolic blood pressure; TNF – tumour necrosis factor.

**Table 3:** Vascular and Laboratory Assessments in HV Arm.

	Placebo		Lycopene		*P*-value*
	Day 1[Mean Value (SE)]	Day 56[Mean Value (SE)]	Day 1[Mean Value (SE)]	Day 56[Mean Value (SE)]	
**Arterial Stiffness**					
Aortic PWV – m/s	7.9 (0.2)	8.1 (0.4)	7.9 (0.4)	8.1 (0.5)	1.0
AIx – %	24.1 (2.9)	23.2 (2.2)	26.3 (2.6)	25.1 (2.8)	0.9
**Blood Pressure**					
Home SBP –mmHg	121 (3)	122 (3)	121 (4)	119 (3)	0.2
Home DBP – mmHg	74 (2)	75 (3)	72 (2)	71 (2)	0.3
Home SBP variability	8.2 (0.9)	7.8 (0.7)	8.3 (0.6)	8.1 (0.7)	0.9
Clinic SBP – mmHg	127 (4)	125 (4)	126 (4)	129 (3)	0.2
Clinic DBP – mmHg	76 (2)	75 (2)	75 (2)	77 (2)	0.2
Central SBP – mmHg	120 (4)	116 (4)	116 (4)	120 (4)	0.1
Central DBP – mmHg	78 (2)	77 (3)	76 (2)	78 (2)	0.2
**Laboratory Markers**					
Lycopene – µg/L	182 (35)	160 (29)	170 (16)	267 (18)	<0.001
LDL – mmol/L	3.89 (0.26)	3.79 (0.21)	3.51 (0.20)	3.45 (0.19)	0.8
HDL – mmol/L	1.52 (0.11)	1.56 (0.08)	1.63 (0.10)	1.68 (0.11)	0.8
hsCRP – mg/L	2.83 (1.15)	1.65 (0.42)	1.15 (0.25)	1.87 (0.39)	0.6
ox-LDL – U/L	50.5 (32.8)	48.5 (22.1)	47.9 (25.1)	46.1 (25.2)	0.9
MMP-9– ng/ml	35.4 (27.2)	36.3 (20.4)	38.6 (17.5)	41.9 (24.7)	0.7
IL-6– pg/ml	0.92 (0.87)	0.84 (0.59)	1.32 (2.86)	1.02 (1.74)	0.8
TNF-α – pg/ml	5.55 (2.88)	5.32 (2.88)	5.39 (2.23)	4.97 (2.10)	0.7
Nitrotyrosine – µM	119.3 (55.5)	145.4 (88.1)	96.1 (35.9)	118.9 (37.1)	0.8

Data are presented as mean values (standard error - SE). *P-value is for overall comparison in delta (day 56 - day 1) values across placebo and lycopene treated groups. AIx – augmentation index; DBP – diastolic blood pressure; HDL – high-density lipoprotein; hsCRP – high sensitivity C-reactive protein; HV – healthy volunteer; IL – interleukin; LDL – low-density lipoprotein; MMP-9– matrix metalloproteinase; ox-LDL – oxidised low-density lipoprotein, PWV – pulse wave velocity; SBP – systolic blood pressure; TNF – tumour necrosis factor.

**Table 4:** Post-hoc comparisons of baseline values between CVD Patients and HV arms.

	CVD Patients [Mean Value (SD)]	HVs [Mean Value (SD)]	*P-*value*
**Arterial Stiffness**			
Aortic PWV – m/s	9.0 (2.9)	7.9 (1.6)	0.05
AIx – %	32.2 (7.0)	27.3 (10.2)	0.02
**Blood Pressure**			
Home SBP – mmHg	126 (12)	121 (13)	0.1
Home DBP – mmHg	76 (10)	72 (8)	0.1
Home SBP variability	9.0 (2.9)	8.2 (2.8)	0.3
Clinic SBP – mmHg	137 (12)	127 (16)	0.003
Clinic DBP – mmHg	81 (8)	77 (9)	0.04
Central SBP – mmHg	129 (12)	118 (17)	0.002
Central DBP – mmHg	81 (8)	77 (9)	0.04
**Laboratory Markers**			
Lycopene – µg/L	140 (76)	174 (94)	0.1
LDL – mmol/L	2.4 (0.7)	3.6 (0.9)	<0.001
HDL – mmol/L	1.3 (0.4)	1.6 (0.5)	0.004
hsCRP – mg/L	1.90 (2.26)	1.73 (2.60)	0.8
ox-LDL – U/L	33.8 (77.8)	48.7 (117.7)	<0.001

Data are presented as mean values (standard deviation - SD). *P-value is for comparison between cardiovascular disease (CVD) patients arm and healthy volunteer (HV) arm at baseline using unpaired, 2-tailed Student *t*-tests. AIx – augmentation index; DBP – diastolic blood pressure; HDL – high-density lipoprotein; hsCRP – high sensitivity C-reactive protein; LDL – low-density lipoprotein; ox-LDL – oxidised low-density lipoprotein, PWV – pulse wave velocity; SBP – systolic blood pressure.

### Blood Pressure

Lycopene treated CVD patients achieved reductions in clinic peripheral and central diastolic blood pressure on day 56 compared to day 1 (peripheral BP 2.9 mmHg lower, 95% CI: −5.5 to −0.2, *P* = 0.03 and central BP 3.3 mmHg lower, 95% CI: −6 to −0.5, *P* = 0.02); but these changes were not significant when compared to placebo. No other changes were observed in BP parameters (**Tables 2** and **3**). In post-hoc analysis, baseline clinic BP and central BP were higher in the CVD arm compared with HVs (**Table 4**). Mean home BPs did not differ between CVD and HVs (**Table 4**).

### Laboratory Assessments

Lycopene-treated patients in the CVD arm showed increases in serum lycopene compared with placebo-treated CVD patients (Δ lycopene 130±80 (active) vs. 50±73 µg/L (placebo), *P* = 0.003). Similarly, serum lycopene levels increased in the HV arm with active treatment when compared to placebo (Δ lycopene 97±82 (active) vs. –19±77 µg/L (placebo), *P*<0.001). Serum lycopene increased by a similar amount for lycopene treated subjects in both the CVD and HV arms of our study (*P* = 0.2). No significant changes were noted in other parameters in any group after lycopene treatment (**Tables 2** and **3**). Urinary isoprostane readings were below the limits of assay detection in over half the patients in both CVD and HV arms of the study and are therefore not reported.

### Safety and Compliance Assessments

Oral lycopene supplementation was safe and well tolerated. There were no serious adverse events, with a higher minor adverse event rate in the placebo arm than the lycopene treated arm (54% vs. 23%, *P* = 0.02). Frequently reported adverse events are shown in **Table S3**; the most common event was gastrointestinal upset and all were classed as mild. There were no differences between lycopene vs. placebo groups for routine biochemical (including liver function tests), haematological, heart rate, blood pressure, or ECG parameters. Overall compliance, as assessed by manual pill count on day 56, was 96% in the CVD arm and 94% in the HV arm.

## Discussion

We have demonstrated that despite optimal secondary prevention medication, endothelial function is impaired in patients with cardiovascular disease, and this is improved by oral supplementation with 7 mg lycopene, without any concomitant changes in traditional risk factors such as BP or lipid profiles, or measures of inflammation. In contrast, we were unable to demonstrate any changes in endothelial function or other parameters after lycopene treatment in HVs.

Complex dietary modifications to alter CVD risk are recognised as being effective but difficult to implement [34]. Recent data from patients with a prior cardiovascular event in the Prospective Urban Rural Epidemiology (PURE) study indicate that adherence to a healthy diet is only apparent in 39% of the 7,519 patients studied [35]. Further, the Prevención con Dieta Mediterránea (PREDIMED) interventional study added to increasing evidence of the benefits of a ‘Mediterranean’ diet including tomato products, in addition to a diet low in saturated fat, both for primary and secondary prevention of CVD [3]–[5]. The specific mechanisms underlying benefits of a healthy diet are poorly defined and although dietary supplements receive much attention within the public arena, there is a paucity of well-conducted mechanistic studies. Previous trials of vitamin C and E supplementation demonstrated conflicting effects on vascular function *in-vivo* [36]–[39], which may explain the negative outcome trials associated with them [40].

As the most potent antioxidant known [6], there is biological plausibility and epidemiological data [9] suggesting that lycopene intake may be at least partly responsible for variations in cardiovascular mortality across Europe. Previous studies investigating the vascular effects of lycopene have provided difficult to interpret evidence of its effects – using unstandardized large volume tomato food-based modes of delivery [13], [15], [18], [19], combining lycopene with other antioxidants thus making interpretation of the relative benefits of individual components in healthy people unclear [16], [17], or using methods of assessing vascular function that are not known to correlate well to clinical outcomes [17].

We opted for a pragmatic, translational study design by selecting a population of stable patients with CVD on statins (which are known to improve endothelial function) [31], and in whom ‘traditional’ risk factors for CVD were clinically optimised. No specific dietetic advice was provided to minimise intentional variations in dietary intake, and we corroborated the benefits of intervention by measuring and demonstrating increased serum lycopene levels in the intervention (lycopene treated) groups. This enabled us to establish a potential benefit for lycopene intervention in a real world setting in addition to optimal secondary prevention treatment for patients with CVD.

Endothelial dysfunction may explain the ‘residual risk’ of further events seen in patients with CVD despite optimal control and treatment of vascular risk factors [20]–[22]. Importantly, we measured endothelial function using forearm plethysmography, which is the gold-standard method of assessing vascular function, and provides not only mechanistic information surrounding nitric oxide bioavailability, but is a surrogate marker for risk of developing CVD events, and improves risk prediction when added to existing risk scores [26]. We were able to demonstrate a 53% improvement in ACh responses post-lycopene therapy in CVD patients (who were impaired at baseline), but not HVs. This is comparable to the effect size seen previously with simvastatin 20 mg in untreated hypercholesterolaemic patients [31], although a novel finding in our study was that these effects were beyond that conferred by effective statin therapy in an atherosclerotic population. Parallel to this, we did not demonstrate any change in SNP responses in either arm, suggesting that this effect was likely primarily due to an augmentation of stimulated NO production to acetylcholine, rather than improvement in smooth muscle sensitivity. The role of NO in maintaining elastic artery stiffness is unclear, with newer evidence suggesting it may not have a significant role [41], [42]. We found no change in measurements of arterial stiffness, and therefore postulate that lycopene’s effects may be predominantly at the level of the smaller vessels, such as resistance arteries, rather than larger vessels. However, we contend that we are unable to exclude if a longer duration of treatment with lycopene, or a higher dose of lycopene may have produced more changes in arterial stiffness.

Existing data suggest lycopene supplementation leads to reductions in BP in subsets of patients with untreated pre-hypertension and a reduction in markers of systemic inflammation in patients with type 2 diabetes; although there are conflicting results in healthy subjects [13], [14], [17], [18], [43]. We did not observe any effect of lycopene on systemic markers of inflammation (hsCRP, cytokine profile), BP, or arterial stiffness. However, both CVD and HV subjects had low hsCRP levels at baseline, suggesting that they were not systemically inflamed. Moreover, the patients with CVD were all receiving statins and had low LDL and ox-LDL levels which may account for the lack of observed effects on systemic inflammatory markers. We noted a small decrease in diastolic BP in lycopene treated CVD patients that was not significant when placebo corrected. Interestingly, a recent large interventional dietary study of the Mediterranean diet in patients at high risk of CVD found decreases in diastolic, but not systolic BP [44].

We did not include any dietary restrictions in our randomised, blinded but real world, pragmatic trial design. Nevertheless, we have demonstrated that subjects in the lycopene group had increased their serum lycopene levels significantly in both CVD and HV arms of our trial, suggesting natural variations in dietary intake were probably minimal in our randomised trial across both lycopene and placebo groups in both arms. Whilst we accept the limitations of a post-hoc correlation analysis combining data from 2 non-stratified arms (namely HV and CVD patients), we were able to demonstrate a correlation between changes in serum lycopene concentrations and changes in endothelium dependent vasodilatation, irrespective of whether this increase in lycopene was due to changes in dietary intake or treatment allocation. This would need further exploration in a prospective manner but does provide some additional evidence to substantiate the mechanisms underlying trials such as the PREDIMED study. Our baseline serum lycopene levels were higher than those found in observational studies from Finland linking lycopene levels to risk of CVD [10], which the authors attribute to a lower dietary intake of lycopene. In their study, increases in serum lycopene across the quartiles (lowest quartile <38 µg/L to highest quartile >129 µg/L) showed decreases in CVD risk [10]. The change in lycopene levels seen in our intervention group (mean lycopene increase in CVD active group 130±8 µg/L) was greater than the interquartile range seen in the observational study, suggesting that even modest increases in serum lycopene may affect endothelial function in atherosclerotic patients with endothelial dysfunction. In a previous study by Kim *et *al. using a tomato extract which also contained a mixture of other antioxidants, the 15 mg ‘lycopene preparation’ increased serum lycopene levels by a mean of 130±10 µg/L, which is almost the same as the increase seen in our CVD arm at a dose of 7 mg using a lycopene-only preparation [17]. However, it was unclear from that particular study which of the antioxidants exerted the effects seen due to the mixed nature of the active intervention.

Lycopene enhances endothelial function in CVD patients independently of ‘traditional’ risk factors (BP, LDL) or systemic inflammation. Little is known about how a ‘direct antioxidant’ effect may improve endothelial function. One hypothesis is that a reduction in reactive oxygen species would increase the bioavailability of NO and potentially decrease DNA and protein damage [45]. Unfortunately, urinary isoprostane levels were below the lower detection limit of our assay for the majority of the samples, and there was wide variation in plasma nitrotyrosine levels, so we were unable to confirm this effect in this study. Although tomato-based dietary studies suggested improvements in oxidative stress *in-vivo* [13], [17], [19], the antioxidant mechanism of action for lycopene is controversial, with some authors suggesting that at the concentrations found in the body, this mechanism is unlikely to be significant [46]. Studies using other antioxidants suggest that structural modifications of the compound due to metabolic transformations *in-vivo* may profoundly affect bioactivity and mechanisms of action [47]. Other putative ‘antioxidants’ have shown beneficial effects on atherosclerosis without effects on markers of oxidative stress so further work to determine the exact mechanism of action of lycopene is needed [48].

Several limitations of the study are worth highlighting. The study was designed as a proof-of-concept study investigating mechanistic actions of lycopene using forearm plethysmography. The study was powered on the primary endpoint of forearm responses to ACh, which may explain why L-NMMA responses did not reach statistical significance in the CVD arm and similarly why other biomarker data was negative in a cohort with optimally controlled risk factors. Lind *et al.* [26] showed that baseline endothelium dependent responses to ACh correlate independently with the risks of future CVD events. Drugs which improve CVD mortality such as statins, β-blockers, ACE-inhibitors, and angiotensin receptor blockers have all shown beneficial effects on endothelial function as measured by forearm responses to ACh [31], [49]–[51], but no study has demonstrated that the reversal of endothelial dysfunction with an active intervention also alters CVD mortality in the same individuals. These mechanistic results must therefore be confirmed with subsequent interventional clinical outcome studies.

The choice of lycopene dose was limited by the availability of different dose regimens so we are unable to be definitive if a higher dose of lycopene may have altered endothelial responses in HVs. The bioavailability of lycopene varies according to the preparation of tomatoes (puree, ketchup etc.), as well as its origin, size, shape, and the manner in which it is consumed (very bioavailable in the presence of oil, for instance). Studies consistently highlight the benefits of a diet high in fruits and vegetables for the secondary prevention of CVD, with more recent studies suggesting a role for olive oil consumed in conjunction with tomato products in primary prevention in patients with CVD risk factors [3]–[5]. Supplementing one component of this diet may not necessarily replace the benefits of a complex mixture of interacting nutrients as part of a healthy diet. However, our study does provide mechanistic evidence for the benefits of one such component of a Mediterranean diet in CVD patients.

## Conclusions

We have demonstrated, in a double blind, randomised, controlled mechanistic trial, that lycopene improves endothelial function in CVD patients who demonstrated impaired function at baseline, despite optimal secondary prevention medication, but not in age-matched, HVs. Our translational, *in-vivo*, physiological study provides a mechanistic explanation for the beneficial effects of lycopene, a component of the Mediterranean diet on the vasculature. It reinforces the need for a healthy diet to augment endothelial function in at-risk populations despite optimal medical therapies. Most importantly, further interventional studies are warranted to determine if lycopene supplementation could alter cardiovascular outcomes in at-risk populations.

## Supporting Information

Figure S1 **Chemical Structures of Selected Antioxidants.** (A) Lycopene showing numerous double bonds; and (B) Vitamin E (α – tocopherol). [Adapted from Di Mascio P, Kaiser S, Sies H (1989) Lycopene as the most efficient biological carotenoid singlet oxygen quencher. Arch Biochem Biophys 274∶532–538].
(TIF)

Table S1 **Mean (SE) FBF Values in CVD Patient Arm.**
(DOCX)

Table S2 **Mean (SE) FBF Values in Healthy Volunteer Arm.**
(DOCX)

Table S3 **Self-reported Adverse Events Profile.**
(DOCX)

Checklist S1 **CONSORT Checklist.**
(DOC)

Protocol S1 **Trial Protocol.**
(DOCX)
