# Supplementary figures and images for: Effects of Oral Lycopene Supplementation on Vascular Function in Patients with Cardiovascular Disease and Healthy Volunteers: A Randomised Controlled Trial
Source: PLoS One. 2014 Jun 9;9(6):e99070. doi: 10.1371/journal.pone.0099070 (PMC4049604; doi:10.1371/journal.pone.0099070)

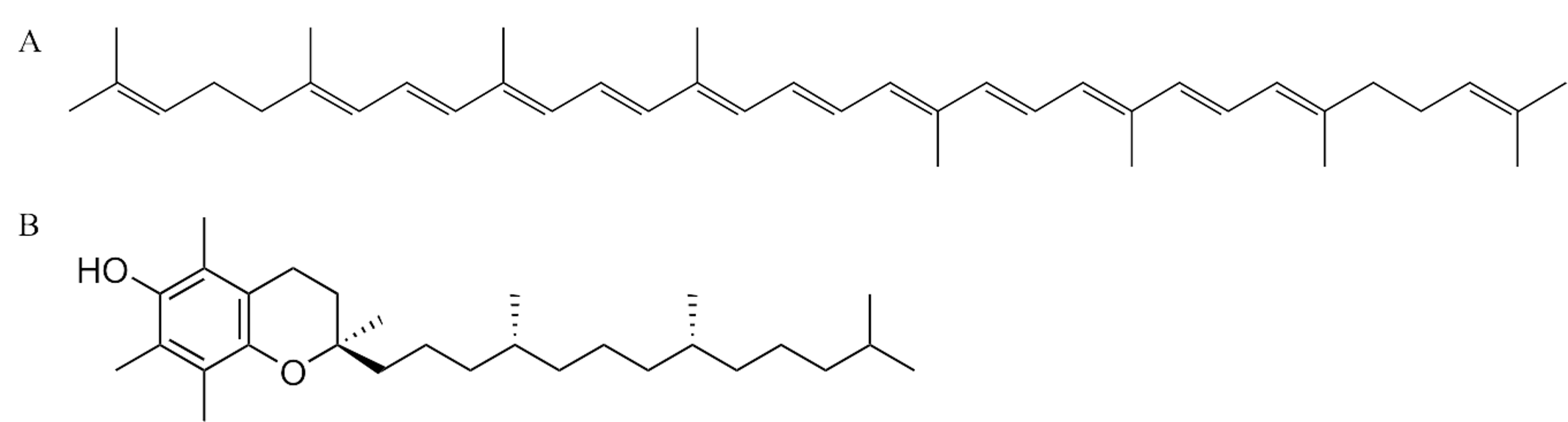

Supplement: Figure S1 — Chemical Structures of Selected Antioxidants. (A) Lycopene showing numerous double bonds; and (B) Vitamin E (α – tocopherol). [Adapted from Di Mascio P, Kaiser S, Sies H (1989) Lycopene as the most efficient biological carotenoid singlet oxygen quencher. Arch Biochem Biophys 274∶532–538]. (TIF) [file pone.0099070.s001.tif]
