# Supplementary material for: Effects of Oral Lycopene Supplementation on Vascular Function in Patients with Cardiovascular Disease and Healthy Volunteers: A Randomised Controlled Trial
Source: PLoS One. 2014 Jun 9;9(6):e99070. doi: 10.1371/journal.pone.0099070 (PMC4049604; doi:10.1371/journal.pone.0099070)
Supplement: Table S2 — Mean (SE) FBF Values in Healthy Volunteer Arm. (DOCX) [file pone.0099070.s003.docx]

**Table S2. Mean (SE) FBF Values in Healthy Volunteer Arm**

|  | **Placebo** | | **Lycopene** | |
| --- | --- | --- | --- | --- |
|  | **Day 1**  (Mean [SE]) | **Day 56**  (Mean [SE]) | **Day 1**  (Mean [SE]) | **Day 56**  (Mean [SE]) |
| **ACh** | | | | |
| Baseline | 1.88 (0.30) | 1.89 (0.24) | 1.96 (0.16) | 1.93 (0.14) |
| ACh 7.5μg | 5.10 (0.77) | 5.19 (0.82) | 6.31 (0.99) | 5.92 (0.65) |
| ACh 15μg | 7.11 (0.93) | 6.59 (1.36) | 7.73 (1.20) | 7.56 (0.88) |
| Overall comparison of lycopene vs. placebo (for day 56 vs. day 1) for ACh 15μg | | | | |
| Treatment Difference (95% CI), % | | | | 38 (-15 to 92) |
| *P* | | | | 0.73 |
| **SNP** | | | | |
| Baseline | 1.92 (0.32) | 2.11 (0.25) | 2.30 (0.23) | 2.21 (0.19) |
| SNP 3μg | 6.95 (0.62) | 8.45 (1.21) | 8.46 (0.84) | 8.35 (0.63) |
| SNP 10μg | 9.72 (1.14) | 12.50 (1.71) | 12.60 (1.09) | 12.36 (0.99) |
| Overall comparison of lycopene vs. placebo (for day 56 vs. day 1) for SNP 10μg | | | | |
| Treatment Difference (95% CI), % | | | | -7 (-43 to 28) |
| *P* | | | | 0.16 |
| **L-NMMA** | | | | |
| Baseline | 2.16 (0.26) | 2.44 (0.39) | 2.83 (0.32) | 2.62 (0.41) |
| L-NMMA 2μmol | 1.72 (0.27) | 1.66 (0.24) | 2.16 (0.21) | 2.11 (0.34) |
| L-NMMA 4μmol | 1.65 (0.31) | 1.30 (0.14) | 1.86 (0.21) | 1.74 (0.19) |
| Overall comparison of lycopene vs. placebo (for day 56 vs. day 1) for L-NMMA 4μmol | | | | |
| Treatment Difference (95% CI), % | | | | -15 (-40 to 8) |
| *P* | | | | 0.38 |

Forearm blood flow (FBF) data are presented as mean (standard error - SE). Comparisons were made using a repeat measures ANOVA with terms for drug/placebo, visit day, infusion dose within day, and interaction of treatment and dose within day, in which baseline saline was treated as infusion dose zero. Data refer to infused arm values in ml/100ml tissue/min. Point estimates of treatment difference (expressed as percentage treatment difference) with corresponding 95% confidence intervals (CI) are shown for the higher doses of acetylcholine (ACh), sodium nitroprusside (SNP), or NG-monomethyl-L-arginine (L-NMMA).
