# Supplementary material for: Effects of Oral Lycopene Supplementation on Vascular Function in Patients with Cardiovascular Disease and Healthy Volunteers: A Randomised Controlled Trial
Source: PLoS One. 2014 Jun 9;9(6):e99070. doi: 10.1371/journal.pone.0099070 (PMC4049604; doi:10.1371/journal.pone.0099070)
Supplement: Table S3 — Self-reported Adverse Events Profile. (DOCX) [file pone.0099070.s004.docx]

**Table S3. Self-reported Adverse Events Profile**

| **System – n** | **Lycopene (n = 48)** | **Placebo (n = 24)** |
| --- | --- | --- |
| Respiratory | 4 | 13 |
| Cardiovascular | 0 | 1 |
| Gastrointestinal | 9 | 8 |
| Neurological | 3 | 5 |
| Musculoskeletal | 1 | 3 |
| Dermatological | 0 | 1 |
| TOTAL | 17 AEs in 11 subjects | 31 AEs in 13 subjects |

Self-reported adverse events (AE) profile across both cardiovascular disease patients and healthy volunteer arms. All AEs were classed as mild.
