# Supplementary material for: Effects of Oral Lycopene Supplementation on Vascular Function in Patients with Cardiovascular Disease and Healthy Volunteers: A Randomised Controlled Trial
Source: PLoS One. 2014 Jun 9;9(6):e99070. doi: 10.1371/journal.pone.0099070 (PMC4049604; doi:10.1371/journal.pone.0099070)
Supplement: Protocol S1 — Trial Protocol. (DOCX) [file pone.0099070.s006.docx]

**‘TomVasc’: Vascular effects of nutritional supplementation with a standardised preparation of tomato extract (Ateronon) *in vivo* in man.**

**Version 7**

**28 November 11**

**Chief Investigator**

Dr Joseph Cheriyan

Box 128, Level 3

ACCI Building

Addenbrooke’s Hospital

Hills Road

Cambridge

CB2 2QQ

tel: 01223 296070

email: jc403@cam.ac.uk

**Protocol Authorised by Chief Investigator**

**Signature:**

**Date:**

**Clinical Queries**

Clinical queries should be directed to Dr Joseph Cheriyan.

**Sponsor**

Cambridge University Hospitals NHS Foundation Trust is the main research sponsor for this study. For further information regarding the sponsorship conditions, please contact the Research Governance Manager at:

Cambridge University Hospitals NHS Foundation Trust,

Box 277,

Hills Road,

Cambridge

CB2 0QQ

01223 348490

R&Denquiries@addenbrookes.nhs.uk

**PROTOCOL**

**Background & Rationale**

Atherosclerosis is a progressive, yet initially asymptomatic disease in which the accumulation of lipids, inflammatory cells and other connective tissue cells result in endothelial dysfunction, arterial stiffness and eventual narrowing or even occlusion of affected arteries after rupture of an inflamed, unstable atheromatous plaque. The incidence of cardiovascular disease varies throughout Europe with a reduced incidence in those areas with a Mediterranean diet which contains a larger proportion of fruit, vegetables and olive oil and has been well published over the last 20 years^1-3^.

Dietary therapies such as the Mediterranean diet have been shown to be associated with a reduced risk of atherosclerotic diseases ^4^ in particular the intake of tomatoes and tomato-based products. The reasons postulated are that such diets are rich in antioxidants. Lycopene is a lipophilic, active carotenoid component of tomatoes – and levels of lycopene in plasma or adipose tissue are inversely correlated to risk of disease. It is one of the most potent antioxidants with a singlet-oxygen quenching ability twice that of β-carotene and 10 times that of α-tocopherol^5^. The bioavailability of lycopene varies according to the preparation of tomatoes – for example it is very bioavailable in the presence of oil. Tomato paste also has enhanced bioavailability compared to raw tomatoes, tomato juice or to tablets/capsules containing highly concentrated tomato extract. Additionally, tomatoes of different origins, sizes, shapes, and the nature in which it is consumed has profound effects on the amount of lycopene it contains. This explains why there is a variable correlation between tomato intake and plasma/adipose lycopene levels and disease risk.

There have been no studies of standardised intake of tomato extract to examine its effect on vascular physiology to date. There are association studies that inversely link lycopene levels with surrogate endpoints of cardiovascular disease or its incidence both in adipose tissue and serum. ^6, 7^

Endothelial function as measured by forearm blood flow (plethysmography) is a well described method of assessing and determining vascular function and can interrogate endothelium dependent and independent mechanisms of nitric oxide bioavailability^8^ and can predict risk of future events^9^. Arterial stiffness is now rapidly being accepted as a measure of functional and structural changes which accompanies cardiovascular disease and is increasingly associated with outcomes^10^.

We would like to determine whether a standardised preparation of tomato extract containing a defined amount of lycopene may have effects on vascular physiology. We wish to investigate the effects of a standardised tomato extract product (using a commercially available nutritional supplement - Ateronon) produced by a high quality food manufacturer on vascular function (endothelial function and arterial stiffness).

We plan to study this in 2 groups of subjects – healthy older people and stable cardiovascular disease patients on stable statin therapy.

**Hypotheses**

1. Dietary supplementation with a standardised tomato extract product improves endothelial function in healthy older people and patients with stable cardiovascular disease.
2. Dietary supplementation with a standardised tomato extract product improves parameters of arterial stiffness.
3. Dietary supplementation with a standardised tomato extract product reduces lipoprotein oxidation and circulating markers of endothelial damage.

**Study Objectives**

Dietary supplementation with a standardised tomato extract product improves endothelial function in healthy older people and patients with stable cardiovascular disease.

**Study Design**

Subjects will be screened, then randomised to receive Ateronon or placebo for 56 days. Randomisation will be unequal for each group (ratio 2:1 drug:placebo). Forearm plethysmography studies will be conducted pre-dose and at the end of the treatment period. Arterial stiffness will also be measured at both study visits.

**Visit 1** **Screening Visit 2 Treatment Visit 3 Follow-up**

**(4 weeks) Randomisation (8 weeks) End of (2 weeks)**

**Treatment**

**Key Assessments (Visits 2 & 3)**

arterial stiffness, forearm blood flow, blood sampling for lipoprotein oxidation and markers of endothelial damage

**‘Healthy’**

- 30-80 years
- Non-smoking
- No hypertension/CVD
- No renal/respiratory/

neurological disease

- No diabetes

24 tomato extract o.d.

12 placebo o.d.

**‘Stable CVD’**

- 40-80 years
- Previous IHD (MI, stent, angina), TIA or ischaemic stroke disease or peripheral vascular disease
- Statin use stable

24 tomato extract o.d.

12 placebo o.d.

**Figure 1.** Study Design

**Number of Participants**

a) Sufficient numbers will be recruited to complete 36 healthy older male or female subjects (‘Healthy Group’)

AND

b) Sufficient numbers will be recruited to complete 36 stable cardiovascular disease patients on stable statin therapy (‘Stable CVD Group’).

Subjects will be asked to continue their intake of tomato and tomato based products as before (*i.e.* not to change their intake whilst on the study) to reduce interference with the study question. Other potential confounding effects of vascular function *e.g.* glucose, lipid profile and blood pressure will also be measured.

**Outcome Measures**

**Primary outcome:**

Forearm blood flow ratio and/or absolute flow in the infused arm (& % change), as measured by venous occlusion plethysmography, in response to intra-arterial acetylcholine infusion.

**Secondary outcomes:**

- Forearm blood flow ratio and/or absolute flow in the infused arm (& % change), as measured by venous occlusion plethysmography, in response to intra-arterial sodium nitroprusside infusion.
- Forearm blood flow ratio and/or absolute flow in the infused arm (& % change), as measured by venous occlusion plethysmography, in response to intra-arterial L-NMMA infusion.
- Augmentation index (an indicator of arterial stiffness) as estimated by radial arterial pulse contours.
- Pulse wave velocity measured between carotid and femoral artery.
- Lipoprotein oxidation/circulating markers: LDL oxidating activity, AtheroAbzymes, CRP, lipoprotein profile, adiponectin, TNF, IL-2, IL-6, endothelial progenitor and endothelial shedded cells, lycopene concentration in serum, urinary isoprostanes
- Safety and tolerability parameters, including physical examination, blood pressure, heart rate, 12-lead electrocardiograms (ECGs), clinical laboratory tests and adverse event reporting.

**Participants**

**Inclusion criteria (Healthy Group)**

- Aged between 30 and 80 years
- Non-smoking (never smoked regularly [ie >5 cigarettes per day] within past 5 years.)

**Exclusion criteria (Healthy Group)**

- Hypertension (Sustained BP>160/100mmHg), or other cardiovascular disease
- Use of a statin within the preceding three months, or at study start
- Significant renal, respiratory or neurological disease
- Diabetes mellitus
- BMI>32 kg/m^2^, BMI<18 kg/m^2^
- Pregnant or taking the combined oral contraceptive pill or hormone replacement therapy
- Use of vasoactive or other medication including regular NSAIDs
- Use of heparin or warfarin
- Allergy to tomato or tomato products
- Lactose intolerance
- Allergy to whey protein or soy protein
- Any concomitant condition that, at the discretion of the investigator, may affect the participant’s ability to complete the study
- Lack of informed consent
- Current involvement in other research studies

**Inclusion criteria (Stable CVD Group)**

- Aged between 40 and 80 years
- Previous ischaemic heart disease (any of previous myocardial infarction, coronary stent, angina diagnosed on angiography/other imaging modality or exercise/stress testing), transient ischaemic attack or stroke disease or peripheral vascular disease
- If taking a statin, dose must have been stable over preceding 2 months.

**Exclusion criteria (Stable CVD Group)**

- Uncontrolled hypertension BP>180/110
- BMI > 35kg/m^2^
- Pregnant or taking the combined oral contraceptive pill or hormone replacement therapy
- Use of heparin or warfarin
- Allergy to tomato or tomato products
- Lactose intolerance
- Allergy to whey protein or soy protein
- History of active malignant cancer (excluding non-melanoma skin cancer) in the past 3 years.
- Any concomitant condition that, at the discretion of the investigator, may affect the participant’s ability to complete the study.
- Lack of informed consent
- Current involvement in other research studies

**Withdrawal Criteria**

Participants will be withdrawn if unable to tolerate study substance.

**Study Visits**

**Screening – Visit 1 (Days -28 to -1)**

Informed consent, medical history, physical examination, height and weight, 12 lead ECG, BP and other vitals, pregnancy testing (urine) for women of child-bearing potential, concomitant medications. Home blood pressure measurements to be taken morning and evening on 7 days between Visit 1 and Visit 2.

**Visit 2 (Day 0)**

Randomisation. BP and vitals, concomitant medications, weight, arterial stiffness measurements, 12 lead ECG, blood sampling, urine sampling, then forearm study. First dose to be taken that evening.

**Days 1 to 55**:

Daily dosing with Ateronon or placebo. Home blood pressure readings to be taken morning and evening on 7 of the 14 days preceding Visit 3.

**Visit 3 (Day 56 +/- 7 days)**

BP and vitals, concomitant medications, weight, arterial stiffness measurements, 12 lead ECG, blood sampling, urine sampling, then forearm study.

**Follow-up (Day 70 +/- 7 days)**

Telephone screen 2 weeks after Visit 3.

# Participant restrictions

# Participants will be required to refrain from caffeine, alcohol and strenuous exercise for 24 hours prior to Visits 2 and 3, and to fast for a minimum of 4 hours prior to these visits. Participants must not take aspirin, vasoactive/blood pressure medication on the day of Visit 2 or 3 until after the visit.

# Methods

# Blood pressure measurement

Blood pressure will be measured in the seated position after 5 minutes rest. A validated machine will be used. Validated home monitors will be issued to subjects for recording home blood pressure. 3 readings will be made and the average of the last 2 will be used for the average. For home blood pressure measurements, participants will record the 2^nd^ and 3^rd^ readings in the participant diary.

**12-lead ECG**

A 12-lead ECG will be recorded with the participant resting supine.

**Blood Sampling**

***Visits 2 & 3***: 50ml of venous blood will be drawn for estimation of plasma lycopene, standard haematology/chemistry (to include urea and electrolytes, hsCRP, full cholesterol profile), endothelial progenitor cells, oxidised LDL, and other biomarkers of inflammation (eg LDL oxidating activity, AtheroAbzymes, CRP, lipoprotein profile, adiponectin, TNF, IL-2, IL-6, endothelial progenitor and endothelial shedded cells).

**Urine Sampling**

A sample of urine will be collected for measurement of isoprostanes.

Arterial stiffness measurements

Measurements will be made using a non-invasive tonometer applied to the carotid, radial and femoral arteries in the seated and supine position for measurement of pulse wave analysis and aortic pulse wave velocity.

# Forearm blood flow studies

Forearm blood flow will be measured simultaneously in both arms using venous occlusion plethysmography with mercury-in-silastic strain gauges electrically calibrated. The laboratory will be quiet and the temperature will be controlled at 23 ± 2OC. A 27 gauge un-mounted steel needle will be inserted into the non-dominant brachial artery under local anaesthesia (1% lignocaine hydrochloride). Saline or drugs will be infused at a constant rate of 1.0 ml/min by means of a constant rate infusion pump. Basal blood flow will be recorded after 30 min of saline infusion. Acetylcholine (7.5 & 15 µg/min) will then be infused intra-arterially at 1 ml/min as a measure of endothelial NO activity. Each dose will be infused for 6 minutes and forearm blood flow will be measured for 3 minutes at the end of each infusion period. The means of these measurements will be used for analysis. After a further 30 min washout with

**SNP**

3 µg /min

**Saline** 0.9%

**Saline** 0.9%

**ACh** 7.5 µg/

min

**L-NMMA**

4 µmol/

min

0 (minutes) 30 36 42 72 78 84 114 120 126 136

needle 15-18 21-24 27-30 33-36 39-42 63-66 69-72 75-78 81-84 105-108 111-114 117-120 123-126 insertion FBF FBF FBF FBF FBF FBF FBF FBF FBF FBF FBF FBF FBF

**BL1 BL2** **BL3 A1 A2 BL4 BL5 S1 S2 BL6 BL7 L1 L2**

**ACh** 15 µg/

min

**SNP** 10 µg /min

**L-NMMA** 2 µmol/

min

**Saline** 0.9%

**Saline** 0.9%

BP/HR BP/HR BP/HR BP/HR BP/HR BP/HR BP/HR

**Figure 2**. Schematic for measurement of forearm blood flow (FBF). Saline is infused to establish a baseline (BL) before infusion of each challenge agent. BL3, BL5 and BL7 are used as the challenge agent-specific baselines for acetylcholine (ACh), sodium nitroprusside (SNP) and N^G^-monomethyl arginine (L-NMMA), respectively. Each dose of challenge agent is infused for 6 minutes, and FBF recorded in both arms over the last 3 minutes of each infusion.

**Saline** 0.9%

saline, the NO donor, sodium nitroprusside (SNP) will be infused intrarterially at 2 doses (3 and 10 µg/min) for 6 minutes each to test for NO-independent mechanisms. Finally, the NO antagonist L-NMMA will be infused after a 30 minute saline washout, again at 2 doses (2 and 4 µmol/min) and FBF measured as before. A final 30 minutes of saline will then be infused. Blood pressure and heart rate will be recorded at baseline, and at the end of each infusion. The total duration of the assessment is 2 hours and 16 minutes.

**Food Supplement Details**

Ateronon™ is a naturally sourced food supplement consisting of the following ingredients:

Tomato extract (lycopene), whey protein (from milk,) soy, microcrystalline cellulose, gelatine, calcium phosphate, silicon dioxide, polysorbate, glyceryl behenate, colours (red iron oxide, titanium dioxide). All ingredients have been certified as food.

**Supplies**

Ateronon and matching placebo capsules (red in colour) will be provided by Cambridge Theranostics Ltd. Upon randomisation, participants will receive sufficient capsules for the whole treatment period.

**Dosing**

One capsule will be taken daily, in the evening with or immediately after food, throughout the 56-day treatment period.

**Study End**

The study will complete when the last participant in either group completes the follow-up telephone screen, or is withdrawn or lost to follow-up.

**Plan of Further Treatment**

Ateronon^TM^, the standardised tomato extract product being investigated in this study, is commercially available. Participants will be informed of this, enabling them to continue taking the supplement after the study if desired, at their own cost.

# Adverse Events

##

**Definitions**

**Adverse Event (AE):** any untoward medical occurrence in a patient or clinical study subject.

**Serious Adverse Event** **(SAE):** any untoward and unexpected medical occurrence or effect that:

- **Results in death**
- **Is life-threatening** – *refers to an event in which the subject was at risk of death at the time of the event; it does not refer to an event which hypothetically might have caused death if it were more severe*
- **Requires hospitalisation, or prolongation of existing inpatients’ hospitalisation**
- **Results in persistent or significant disability or incapacity**
- **Is a congenital anomaly or birth defect**

Medical judgement will be exercised in deciding whether an AE is serious in other situations. Important AEs that are not immediately life-threatening or do not result in death or hospitalisation but may jeopardise the subject or may require intervention to prevent one of the other outcomes listed in the definition above, should also be considered serious.

Hospitalisations for elective treatment of a pre-existing condition do not need reporting as SAEs.

##

**Reporting Procedures**

All adverse events will be reported. Depending on the nature of the event the reporting procedures below will be followed.

**Non serious AEs**

All such events, whether expected or not, will be recorded.

**Serious AEs**

An SAE form will be completed within 24 hours of the research team becoming aware of the event.

All SAEs will be reported to the Hertfordshire REC where in the opinion of the Chief Investigator, the event was:

- ‘related’, *ie* resulted from the administration of any of the research procedures; and
- ‘unexpected’, *ie* an event that is not listed in the protocol as an expected occurrence

Reports of related and unexpected SAEs will be submitted within 15 days of the Chief Investigator becoming aware of the event, using the COREC SAE form for non-IMP studies.

#### Statistics & Data Analysis

#### Data analysis

The primary outcome measure for the intra-arterial infusions studies will be percentage change in the forearm blood flow ratio and/or absolute flow in the infused arm. Absolute forearm blood flow in each arm, blood pressure and heart rate will also be reported.

Significance will be determined using ANOVA and paired Student’s t tests, corrected for multiple comparisons where indicated.

#### Power calculations

In each group (i.e. healthy older or stable CVD), up to 30 subjects will provide 90% power (P<0.05, 2 sided) to detect a 20% change in forearm blood flow (SD 0.25, α=0.05).

**Interim Analysis**

After 15 participants in each group are studied, an interim analysis will be conducted to determine if a further 21 participants/group are required to be studied.

# Regulatory Issues

**Non-CTIMP Status**

A previous version of this protocol (Version 1, dated 6 July 2009) was submitted to the MHRA, who advised that this research project does not constitute a Clinical Trial of an Investigational Medicinal Product (IMP) as defined by the EU Directive 2001/20/EC.

**Ethical Approval**

The Chief Investigator has obtained approval from the Hertfordshire Research Ethics Committee. The study has been submitted for Site Specific Assessment (SSA) at Cambridge University Hospitals NHS Foundation Trust. The study will be conducted in accordance with the recommendations for physicians involved in research on human subjects adopted by the 18th World Medical Assembly, Helsinki 1964 and later revisions.

**Consent**

Informed consent to enter the study will be sought from each participant only after a full explanation has been given, an information leaflet offered and time allowed for consideration. Signed participant consent will be obtained. The right of the participant to refuse to participate without giving reasons will be respected. All participants are free to withdraw at any time from the protocol treatment without giving reasons and without prejudicing further treatment.

**Confidentiality**

The Chief Investigator will preserve the confidentiality of participants taking part in the study and is registered under the Data Protection Act.

**Indemnity**

NHS Indemnity will apply to any claims made against Cambridge University Hospitals NHS Foundation Trust for negligent harm. There is no cover for non-negligent harm.

##

**Sponsor**

Cambridge University Hospitals NHS Foundation Trust will act as sponsor for this study.

**Funding**

This study is funded from Clinical Pharmacology department funds.

**Audits**

The study may be subject to inspection and audit by Cambridge University Hospitals NHS Foundation Trust under their remit as sponsor and other regulatory bodies to ensure adherence to GCP and the NHS Research Governance Framework for Health and Social Care (2^nd^ edition).

Reference List

(1) de LM, Salen P, Martin JL, Monjaud I, Delaye J, Mamelle N. Mediterranean diet, traditional risk factors, and the rate of cardiovascular complications after myocardial infarction: final report of the Lyon Diet Heart Study. *Circulation* 1999 February 16;99(6):779-85.

(2) Willett WC, Sacks F, Trichopoulou A et al. Mediterranean diet pyramid: a cultural model for healthy eating. *Am J Clin Nutr* 1995 June;61(6 Suppl):1402S-6S.

(3) Serra-Majem L, Roman B, Estruch R. Scientific evidence of interventions using the Mediterranean diet: a systematic review. *Nutr Rev* 2006 February;64(2 Pt 2):S27-S47.

(4) Lockheart MS, Steffen LM, Rebnord HM et al. Dietary patterns, food groups and myocardial infarction: a case-control study. *Br J Nutr* 2007 August;98(2):380-7.

(5) Di MP, Kaiser S, Sies H. Lycopene as the most efficient biological carotenoid singlet oxygen quencher. *Arch Biochem Biophys* 1989 November 1;274(2):532-8.

(6) Kohlmeier L, Kark JD, Gomez-Gracia E et al. Lycopene and myocardial infarction risk in the EURAMIC Study. *Am J Epidemiol* 1997 October 15;146(8):618-26.

(7) Rissanen TH, Voutilainen S, Nyyssonen K, Salonen R, Kaplan GA, Salonen JT. Serum lycopene concentrations and carotid atherosclerosis: the Kuopio Ischaemic Heart Disease Risk Factor Study. *Am J Clin Nutr* 2003 January;77(1):133-8.

(8) Wilkinson IB, Webb DJ. Venous occlusion plethysmography in cardiovascular research: methodology and clinical applications. *Br J Clin Pharmacol* 2001 December;52(6):631-46.

(9) Heitzer T, Schlinzig T, Krohn K, Meinertz T, Munzel T. Endothelial dysfunction, oxidative stress, and risk of cardiovascular events in patients with coronary artery disease. *Circulation* 2001 November 27;104(22):2673-8.

(10) Laurent S, Cockcroft J, Van BL et al. Expert consensus document on arterial stiffness: methodological issues and clinical applications. *Eur Heart J* 2006 November;27(21):2588-605.

# Appendix 1. Summary of investigations, treatment and assessments

|  | **VISIT 1**  Screening  (Day -28 to Day -1) | **VISIT 2**  Randomisation  (Day 0) | **VISIT 3**  End of Treatment  (Day 56) | **FOLLOW-UP**  (telephone screen)  (Day 70) |
| --- | --- | --- | --- | --- |
| Informed Consent | X |  |  |  |
| Medical History | X |  |  |  |
| Physical Examination | X |  |  |  |
| Height | X |  |  |  |
| Weight | X | X | X |  |
| Blood Tests |  | X^[[1]](#footnote-1)^ | X1 |  |
| Urine pregnancy test (women of CBP) | X |  |  |  |
| Urine test (isoprostanes) |  | X | X |  |
| 12-lead ECG | X | X | X |  |
| Blood pressure & vitals | X | X | X |  |
| Arterial stiffness measurements |  | X | X |  |
| Inclusion/exclusion criteria checks | X | X |  |  |
| Randomisation |  | X |  |  |
| Provision of study medication |  | X |  |  |
| Forearm blood flow studies |  | X | X |  |
| Adverse Events |  | X | X | X |
| Concomitant Medications | X | X | X |  |

1. 50ml of venous blood will be drawn for estimation of plasma lycopene, standard haematology/chemistry (to include urea and electrolytes, hsCRP, full cholesterol profile), endothelial progenitor cells, oxidised LDL, and other biomarkers of inflammation (eg LDL oxidating activity, AtheroAbzymes, CRP, lipoprotein profile, adiponectin, TNF, IL-2, IL-6, Endothelial Progenitor and Endothelial Shedded Cells). [↑](#footnote-ref-1)
